# Evaluation of the Solid Dispersion System Engineered from Mesoporous Silica and Polymers for the Poorly Water Soluble Drug Indomethacin: In Vitro and In Vivo

**DOI:** 10.3390/pharmaceutics12020144

**Published:** 2020-02-10

**Authors:** Ziyue Xi, Wei Zhang, Yali Fei, Mingshu Cui, Luyao Xie, Lu Chen, Lu Xu

**Affiliations:** School of Pharmacy, Shenyang Pharmaceutical University, Shenyang 110016, China; lnxiziyue@163.com (Z.X.); zhangwei9501@126.com (W.Z.); f18309806223@126.com (Y.F.); cui_mingshu@126.com (M.C.); xieluyao0221@163.com (L.X.); chenlu182125@163.com (L.C.)

**Keywords:** indomethacin, solid dispersion, mesoporous silica nanoparticles, solubility, bioavailability

## Abstract

This work explored absorption efficacy via an in vivo imaging system and parallel artificial membrane penetration in indomethacin (IMC) solid dispersion (SD) systems. Two different polymer excipients—hydroxypropyl methylcellulose (HPMC) and Kollicoat IR as precipitation inhibitors (PIs)—combined with mesoporous silica nanoparticles (MSNs) as carriers were investigated. The IMC–SDs were prepared using the solvent evaporation method and characterized by solubility analysis, infrared (IR) spectroscopy, powder X-ray diffraction (PXRD), field emission scanning electron microscopy (FESEM), and differential scanning calorimetry (DSC). It was confirmed that IMC successfully changed into an amorphous state after loading into the designed carriers. The in vitro release and stability experiments were conducted to examine the in vitro dissolution rates of IMC–SDs combined with HPMC and Kollicoat IR as PIs which both improved approximately three-fold to that of the pure drug. Finally, in vivo studies and in vitro parallel artificial membrane penetration (PAMPA) experiments ensured the greater ability of enhancing the dissolution rates of pure IMC in the gastrointestinal tract by oral delivery. In brief, this study highlights the prominent role of HPMC and Kollicoat IR as PIs in MSN SD systems in improving the bioavailability and gastrointestinal oral absorption efficiency of indomethacin.

## 1. Introduction

Biopharmaceutics classification system (BCS) Class II drugs are characterized by low solubility and high membrane permeability, so that the dissolution of drugs from dosage formulation is the ultimate rate-limiting step [1], resulting in high permeability but low bioavailability. Many strategies have been applied to improve the dissolution rate and bioavailability of active pharmaceutical ingredients (APIs) including formulation changes, industry strategies, method optimum, etc. [2]. A widely investigated method to circumvent solubility/dissolution-limited bioavailability is to change crystalline drugs into amorphous forms. The optimum ability of the amorphous state is to generate supersaturation solutions possessing high internal free energy to increase the solubility and dissolution rate of insoluble drugs. However, the amorphous state is often rapidly converted back to the crystalline state triggered by thermodynamic force during storage [3]. Precipitation inhibitors (PIs) play a key role when amorphous form crystalline drugs change from the amorphous form to a crystalline state in solution. PIs will prevent the recrystallization trend and help to maintain the supersaturation state after dissolution (spring and parachute effect) [4] via hydrogen bonding interaction. Polymers, including hydroxypropyl methylcellulose (HPMC) and hydroxypropyl methylcellulose acetate succinate (HPMCAS), displayed a superior effect on drug delivery and PIs. Van Speybroeck et al. [5] were the first to combine itraconazole-loaded silica with HPMC as PIs which showed enhanced solubility in vitro and improved bioavailability in vivo. Laine et al. [4] developed a novel supersaturable amorphous formulation through the co-loading of a poorly soluble drug, celecoxib (CXB), and HPMCAS, as a PI, onto silica. The addition of HPMCAS did not reduce the solubility but promoted the complete solid-state conversion of the drug [6]. However, the specific mechanism studies and oral absorption studies, such as gastrointestinal tract absorption of solid dispersions, are still rare.

**Figure 1:**
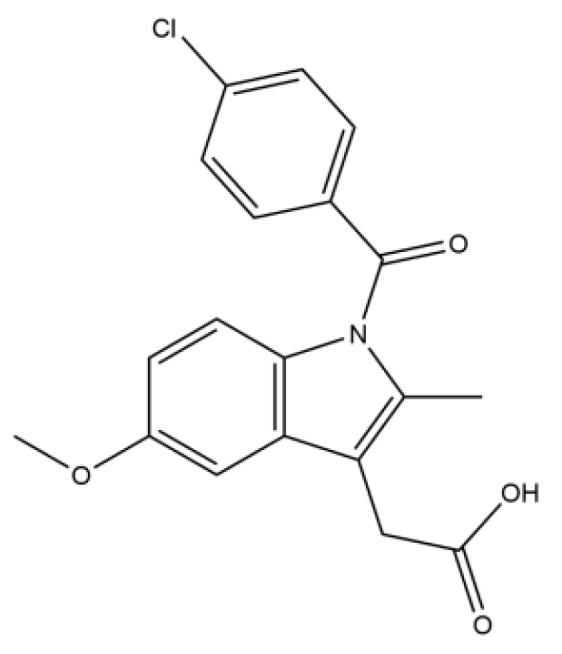
Chemical structures of pure indomethacin.

Indomethacin (IMC, Figure 1) is a non-steroidal anti-inflammatory drug used widely for relieving symptoms of fever, rheumatoid arthritis, cardiovascular and Alzheimer diseases, etc. [7]. However, the application of IMC was limited, because it belongs to BCS Class II drugs. Many attempts have been reported to increase the solubility and dissolution rate of IMC by different formulations and methods in order to reduce adverse reactions and administration frequency [8]. Over the past decades, mesoporous silica nanoparticles (MSNs) have been widely used to ameliorate the dissolution ability of insoluble drugs as drug carriers due to the fact of their unique advantages such as large specific surface area, high pore volume, adjustable pore size, and considerable drug loading capacity [9,10,11,12]. When a drug is loaded into the mesoporous silica pore channels, crystallization is greatly hindered and essentially suppressed, since the long-range ordering associated with crystallization cannot extend beyond the pore diameter which is very important for improving the dissolution rate of poorly soluble drugs [13,14]. However, the release of drugs from MSNs in a supersaturated state may not be sufficient for optimal in vivo performance, because rapid precipitation into a more energy efficient but insoluble form may jeopardize absorption [15]. When PIs are added into a solid dispersion (SD) system, the supersaturation of IMC is maintained for a long time, so that there will be enough IMC dissolved in vivo, resulting in its enhanced bioavailability. Therefore, in order to make insoluble drugs play an effective role in vitro and in vivo, PIs should be added to improve the dissolution rate and oral absorption efficiency.

In this study, we prepared IMC–SDs by the solvent evaporation method using MSNs as carriers and HPMC or Kollicoat IR as PIs. The reason for selecting HPMC and Kollicoat IR was that they are common excipients in pharmaceutical research and also general polymers as SD carriers, and it is unknown if there are differences when they are used as PIs rather than polymer carriers in SD systems. Kollicoat IR is a relatively new polymer in PI studies, and we were very curious whether the efficiency of Kollicoat IR would be greater than HPMC. In addition, it is worth mentioning that Kollicoat IR was first used as a PI. The morphology of MSNs is characterized by transmission electron microscopy (TEM) and nitrogen adsorption/desorption. Powder X-ray diffraction (PXRD), field emission scanning electron microscopy (FESEM), and differential scanning calorimetry (DSC) were used to identify the solid state of IMC. Furthermore, we evaluated the solubilization ability of IMC–SDs added by PIs compared to no PIs and pure IMC by the in vitro drug release experiment. Finally, the pharmacokinetic study was conducted to calculate the in vivo bioavailability of SDs, and the oral absorption of SDs was also investigated by in vivo image system (IVIS).

## 2. Materials and Methods 

### 2.1. Materials

Indomethacin with a purity of more than 99% was purchased from Aladdin Chemical Reagent Corporation, Shanghai, China; Kollicoat IR was obtained from BASF, Port Ludwig, Germany; hydroxypropyl methylcellulose E5 (HPMC) was obtained from Sunhere Pharmaceutical Excipients Chemical Reagent Corporation, Anhui, Huainan, China; Hexadecyl trimethyl ammonium Bromide (CTAB), tetraethyl orthosilicate (TEOS), and 3-aminopropyltriethoxysilane (APTES) were supplied by Yu Wang Chemical Reagent Corporation, Shangdong, Yucheng, China. The IR 820 was provided by Aladdin Chemical Reagent Corporation. Other chemical agents were obtained from Yu Wang Chemical Reagent Corporation, and solvents were of reagent grade and used without further purification. 

### 2.2. Preparation of Mesoporous Silica Nanoparticles (MSNs)

The MSNs were synthesized according to a previously reported method [16]. In brief, the MSNs were synthesized by mixing 380 mL of NaOH solution (0.5 mg/mL) and 100 mL of CTAB solution. Then, 5 mL of tetraethyl orthosilicate (TEOS) was added into the mixture solution drop by drop, and the mixture was stirred at 80 °C for 2 h. Next, the mixture of silica precursor and 5 mL of 10 mol/L HCl solution was stirred at 80 °C for 12 h to remove the CTAB. Finally, the MSN solution was dispersed in 50 mL ethanol.

### 2.3. Preparation of Solid Dispersions

Solid dispersions were prepared via the solvent evaporation method [17]. The IMC and carriers in different ratios were completely dissolved in 5 mL ethanol by stirring at 25 °C, and the weight ratios of IMC to excipients are listed in Table 1. The above solution was mixed uniformly, and organic solvent was removed by rotary evaporation. The resulting substance was dried in a vacuum oven for 1 h at 80 °C, grinded carefully, and sieved (60 mesh, 250 μm) to obtain IMC–SDs.

### 2.4. Characterization of Mesoporous Silica Nanoparticles

The shape of MSNs was imaged by TEM. The surface features, including specific surface area, pore size distributions, and the total pore volume (Vt), were studied by determining the nitrogen adsorption and desorption isotherms using a V-Sorb 2800P surface area and pore size analyzer (Gold App, Liaoning, China). The TEM images were collected using a JSM-6510A (JEOL, Tokyo, Japan) with an accelerating voltage of 200 kV. The pore size was calculated from desorption branches of isotherms using the Barrrett–Joyner–Halenda (BJH) method, while the surface areas were calculated by the Brunauer–Emmett–Teller (BET) method [11].

### 2.5. Characterization of Solid Dispersions 

#### 2.5.1. Field Emission Scanning Electron Microscope (FESEM)

The topographies of the pure IMC and many kinds of IMC–SDs were observed using a field emission scanning electron microscope (ULTRA-PLUS, Zeiss, Jena, Germany). The powders were fixed to a brass specimen holder using double-sided adhesive tape and were made electrically conductive by coating with gold (6 nm/min) in vacuum (6 Pa) using a Zeiss Ion Sputter (E-1030) for 300 s at 15 mA.

#### 2.5.2. Differential Scanning Calorimetry (DSC)

Thermal analysis of the IMC–SDs was performed using a differential scanning calorimeter (Q1000, TA Instruments, New Castle, DE, USA). Approximately 5 mg of a sample was placed in an aluminum pan and heated from 60 to 280 °C at 5 °C/min to obtain DSC curves.

#### 2.5.3. Powder X-ray Diffraction (PXRD)

Powder X-ray diffraction (PXRD) patterns were recorded using a powder X-ray diffractometer (Rigaku Smart Lab, Tokyo, Japan) in a 2θ range of 5–55° with a step width of 0.02° and a scan speed of 2 s per step at 25 °C [6].

#### 2.5.4. Infrared (IR) Spectroscopy

The IR investigations were obtained using an IR spectrometer (IFS55, Bruker, Billerica, MA, USA). The samples of F6, F9, and the corresponding physical mixtures were prepared with potassium bromide and scanned in the region of 400–4000 cm^−1^.

### 2.6. In Vitro Studies

#### 2.6.1. Liquid–Liquid Phase Separation (LLPS) Concentration Determination

The onset LLPS concentration of IMC was determined by ultraviolet (UV) spectroscopy (756 PC UV-2200, Shanghai, China). Methanol solution of IMC (5 mg/mL) was dropwise added into 15 mL of pH 6.8 PBS using a 1 mL injection syringe, after which the solution was pre-equilibrated at 25 °C and stirred at 300 rpm. The LLPS onset concentration was determined at a wavelength of 320 nm using UV spectroscopy by comparison with a standard curve. Triplicate experiments were conducted.

#### 2.6.2. Solubility Studies 

The “crystalline solubility” was measured by the following method. An excess amount of crystal IMC powder was added to 10 mL of pH 6.8 PBS. These mixtures were kept at 25 °C and stirred at 300 rpm for 48 h. Ultracentrifugation was performed at 12,000 rpm and 25 °C for 30 min to separate excess solids using an Optima L-100XP ultracentrifuge (Beckman Coulter Inc., Brea, CA, USA). Then 5 mL of the supernatant was taken for ultraviolet (UV) spectroscopy analysis. All samples were prepared in triplicate.

The solubility of the amorphous IMC in the presence of excipients was also determined by calculating the solution concentration evolved from SDs at 25 °C after 48 h. Briefly, IMC–SDs were prepared by solvent evaporation directly from scintillation vials using a rotary evaporator. Then, 10 mL of pH 6.8 PBS was added. Solution concentration measurements were conducted following the ultraviolet (UV) spectroscopy analysis. The concentration of IMC was calculated with a standard curve built before.

#### 2.6.3. In Vitro Dissolution Studies and Determination of the Optimum Proportion of Solid Dispersion Carriers

The optimum ratios of all solid dispersion carriers were selected by drug release experiments in vitro. All of the weight ratios of IMC to excipients are listed in Table 1. In vitro dissolution studies on pure IMC and SDs with different carriers were carried out using the United States Pharmacopeia Apparatus II (USP II) paddle method (100 rpm, 37 °C, and 250 mL pH 6.8 phosphate buffer solutions) with a ZRS-8G dissolution tester (Shanghai, China) [18]. Ten milligrams of sample powder was placed in the dissolution medium, and 5 mL of pH 6.8 phosphate buffer solution (PBS, artificial intestinal fluid) was withdrawn at pre-designed time intervals (0, 5, 10, 15, 20, 30, 40, 50, 60 and 90 min) prior to filtration. The concentration of samples was analyzed using an UV spectrophotometer (756 PC UV-2200, Shanghai, China) at 320 nm. All measurements were repeated three times.

#### 2.6.4. Moisture Sorption Analysis

Moisture sorption profiles were measured by recording the weight change of SDs under different relative humidity (RH) levels. Powdered amorphous material (IMC 1 mg) was dried at 100 °C until arriving at 0% RH in the experiment and then maintained 0% RH for 1 h. After drying, samples were exposed to relative humidity ranging from 10% to 90% including 18.0%, 32.0%, 42.0%, 59.7%, 79.3%, and 88.0% for 3 h.

For further prediction, the following equation was used to calculate the moisture sorption:

𝑀_𝑎𝑑𝑑𝑖𝑡𝑖𝑣𝑒_ = 𝑀_W_ − 𝑀_D_ (1)

where 𝑀_𝑎𝑑𝑑𝑖𝑡𝑖𝑣𝑒_ is the weight gain of pure IMC or SDs and 𝑀_W_ and 𝑀_𝐷_ are the weights before and after drying, respectively.

#### 2.6.5. Stability Studies

The SDs that were freshly prepared and stored for two months at 25 °C and 60% relative humidity (RH), including IMC–MSNs (F3), IMC–MSNs@Kollicoat IR (F6), and IMC–MSNs@HPMC (F9), were tested by in vitro dissolution again to evaluate the storage stability of the IMC–SDs. The methodology here was the same as that in Section 2.6.1. All measurements were repeated three times.

#### 2.6.6. Parallel Artificial Membrane Penetration (PAMPA)

Parallel artificial membrane penetration is a method for predicting passive absorption of the intestine [19] which was performed in a 12 well Transwell (polycarbonate membrane, 12 mm i.d.). The ability of the compound to diffuse from the donor chamber to the receptor chamber was evaluated by placing a polycarbonate membrane filter between the two chambers. The donor chamber should be pre-treated with a model lipid containing an organic solvent. One milliliter of PBS 6.8 was added to each well of the receptor plate. Then, 0.5 mL of 5 mg/mL IMC or SDs containing the donor solution was added to each well of the donor plate. The samples were shaken at 200 rpm for 0.5, 1.0, 1.5, 2.0, 2.5, 3.0, and 3.5 h at 37 °C, and 200 μL of the solution was taken from the wells of the donor plate at each time point to determine the absorbance, followed by supplementation with 200 μL of pH 6.8 PBS [20].

The test and data processing were carried out according to the product manual [19,21]. The experiment was performed in triplicate, and the results are represented as the mean ± SD. The permeability coefficient (P_eff_) was calculated using Equations (2) and (3).

(2) $$P_{eff}=-\ln\left(1-C_{R}/C_{eq}\right)/S\times \left(1/V_{D}+1/V_{R}\right)\times T_{i}$$

(3) $$C_{eq}=\left(C_{D}\times V_{D}+\;C_{R}\times V_{R}\right)/\left(V_{D}+V_{R}\right)$$

where *P_eff_* is permeability (cm·s^−1^), *S* is the effective filter area (cm^2^), *C_R_* is the concentration in the receptor plate, *V_R_* is the volume of the receptor plate (mL), *C_D_* is the concentration in the donor plate, *V_D_* is volume of the donor plate (mL), and *T_i_* is the incubation time (s).

### 2.7. In Vivo Studies

#### 2.7.1. In Vivo Pharmacokinetic Study

Male Sprague–Dawley rats (body weight 200 ± 20 g) were randomly divided into three groups (*n* = 3). Sprague–Dawley rats (200 ± 20 g) were obtained from Animal Experiment Center of Shanghai Institute of Material Medica, Shanghai, China. All animal experiments were performed by strictly following Institutional Animal Care and Use Committee (IACUC) guidelines (71044, 10 August 2019). Prior to the experiments, the rats were fasted overnight with free access to water. Aqueous suspensions of IMC, SDs with MSNs (F3), SDs with MSNs and Kollicoat IR (F6), and SDs with MSNs and HPMC (F9) at 40 mg/kg were orally administered, respectively, and blood samples (0.5 mL) were collected at different time points (0, 0.5, 1, 2, 4, 6, 8, 12, and 24 h) in microcentrifuge tubes containing heparin by retro-orbital venipuncture technique. The blood samples were immediately centrifuged at 4000 rpm for 10 min, and the supernatant was collected for further processing.

Plasma samples were processed as follows: 200 µL plasma samples were mixed with 20 µL of an internal standard solution (0.5 mg/mL naproxen), 90 µL 10% KH_2_PO_4_, and 1 mL dichloromethane, and then the mixture was vortexed for 3 min. Then, the mixture solutions were centrifuged at 10,000 rpm for 4 min, the organic layer was remained, and then the organic layer was evaporated in a gentle stream of nitrogen at room temperature. The residue was suspended in 100 µL of the mobile phase (acetonitrile:0.01 M H_3_PO_4_ = 55:45). After vortex mixing and centrifugation, samples were analyzed using HPLC.

#### 2.7.2. In Vivo Oral Delivery Imaging Studies

The IMC–SDs labeled IR820 were prepared with 10 mg MSNs and polymers in different ratios as carriers. Male Balb/c mice were administrated intragastrically with pH 6.8 PBS, IMC–MSNs@Kollicoat IR (F6), and IMC–MSNs@HPMC (F9) at a dose of 0.5 mg/kg, respectively. Mice were fasted for 12 h before administration. Afterwards, the mice were tested for fluorescence imaging with an IVIS spectrum imaging system (Perkin Elmer) at excitation and emission wavelengths of 710 nm and 820 nm at 0, 0.5, 1, 2, 4, and 6 h. Meanwhile, the mice were sacrificed and organs, including stomach and small intestine, were taken out for fluorescence imaging at 2 and 6 h, respectively. Images and the fluorescence results were analyzed using Living Image^®^ software. The total radiant efficiency ((p/s)/(µW/cm^2^)) was normalized across images. 

### 2.8. Statistical Analysis

All data were analyzed using GraphPad Prism 7 (GraphPad Software, San Diego, CA, USA) with two-tailed Student’s *t*-tests. All experiments were performed in triplicate unless otherwise mentioned. Error bars used in this work are SD. A *p*-value *<* 0.05 is considered statistically significant. 

## 3. Results and Discussion

### 3.1. Characterization of Mesoporous Silica Nanoparticles (MSNs)

**Figure 2:**
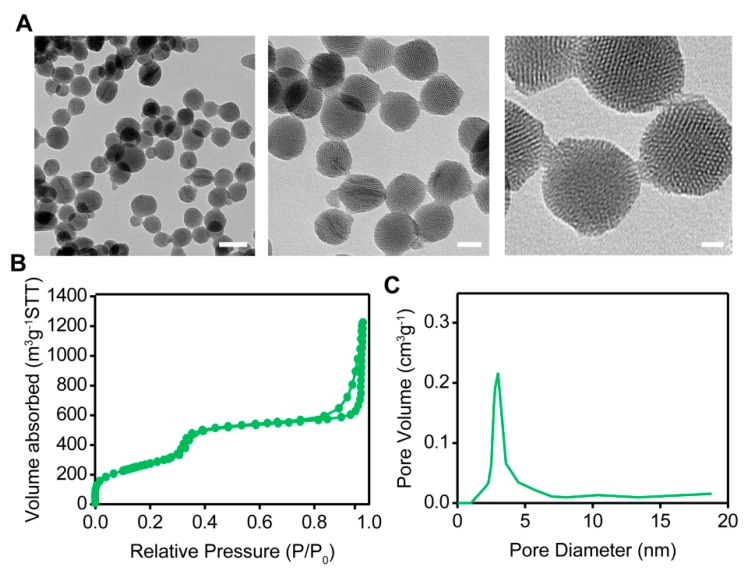
(**A**) TEM images of MSNs. From left to right, the scale bars are 250 nm, 100 nm, and 10 nm, respectively. (**B**) Nitrogen adsorption/desorption isotherm and (**C**) pore size distribution curve of the MSNs.

The TEM images (Figure 2A) indicate that the MSNs were spherical nanoparticles with well-ordered channels. Nitrogen adsorption/desorption isotherms and pore size distribution curves of MSNs are presented in Figure 2B. The obtained curves were analyzed by following reports. The BET reports indicate that the specific surface area of the MSNs was approximately 974.46 m^2^/g, the total pore volume was 0.78 cm^3^/g, and the average pore size was 3.00 nm. These data confirm that we successfully prepared mesoporous silica spherical nanoparticles of uniform size with a relatively narrow pore size distribution. 

### 3.2. Characterization of Solid Dispersions

#### 3.2.1. Field Emission Scanning Electron Microscopy (FESEM)

**Figure 3:**
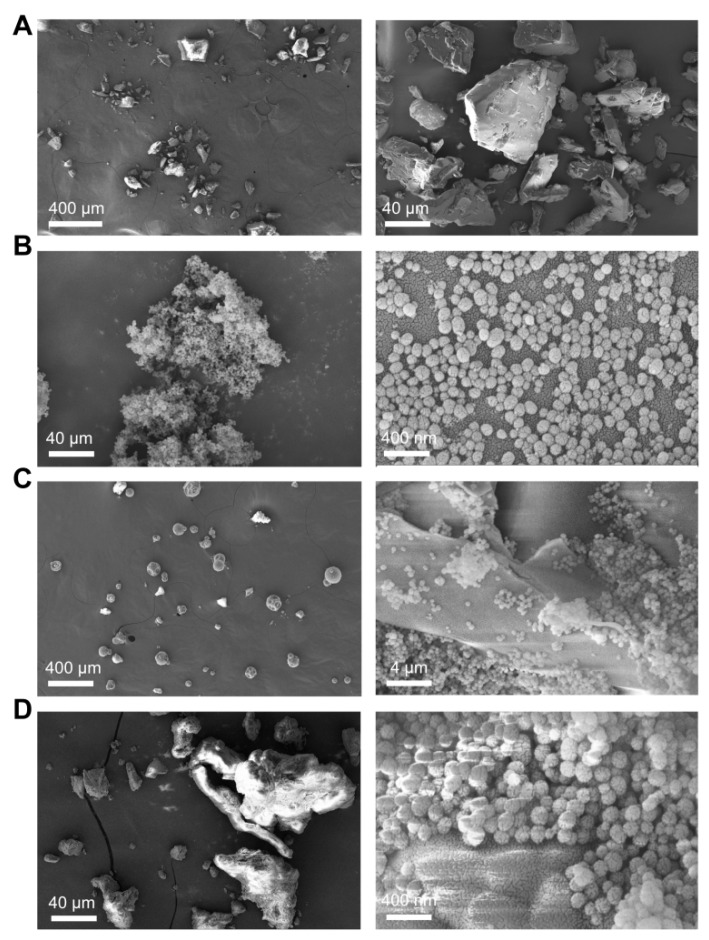
FESEM images of (**A**) pure IMC, (**B**) IMC–MSN (F3), (**C**) IMC–MSN@Kollicoat IR (F6), and (**D**) IMC–MSN@HPMC (F9). Scale bars are as shown in the images.

Powder has many properties, including a crystalline state, flow ability, and adhesion and dissolution rates, which are affected by the surface morphology and shape of powder. The FESEM images are shown in Figure 3, exhibiting clearly the dispersion state of pure IMC and SDs. The shape of pure drug particles is observed in Figure 3A, and the pure IMC was regarded as a “crystalline form” because of its natural crystalline state with a particle size varying from 20 to 100 µm [22]. The SEM image of pure IMC showed a slightly smooth surface and plate-shaped particles (Figure 3A), whereas that of F3 (Figure 3B), F6 (Figure 3C), and F9 (Figure 3D) showed spherical-shaped, smooth-surfaced SDs.

#### 3.2.2. Powder X-ray Diffraction (PXRD), Differential Scanning Calorimetry (DSC), and Thermal Analysis

**Figure 4:**
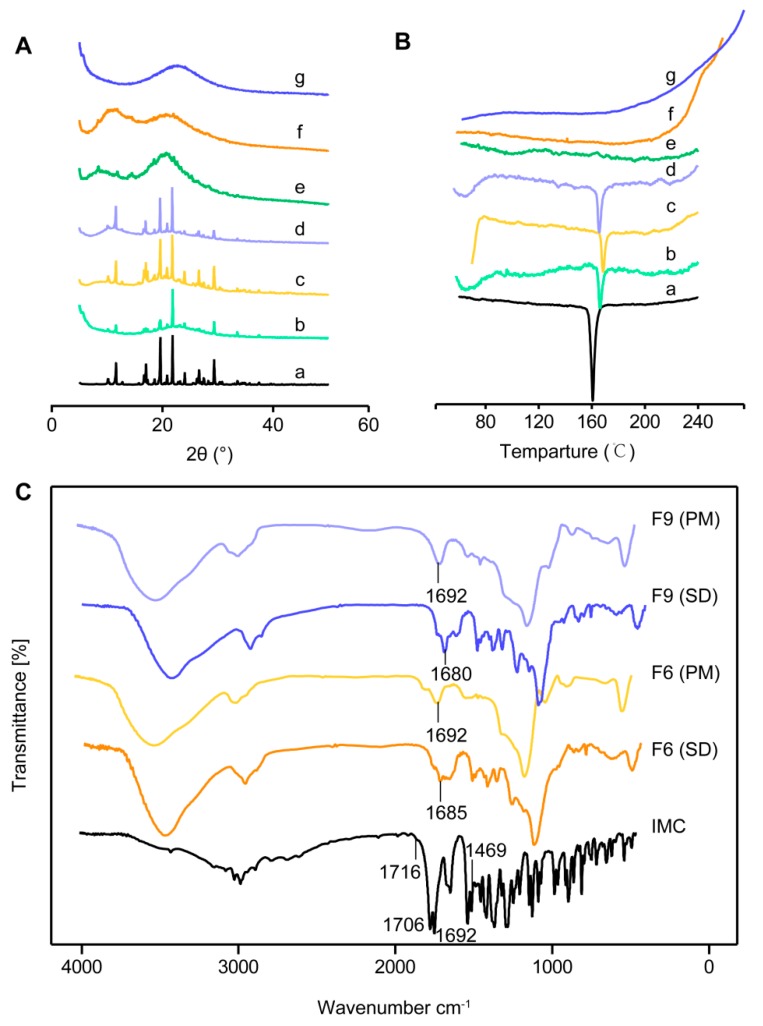
(**A**) PXRD patterns and (**B**) DSC curves of (a) IMC; (b) physical mixture (PM) of MSNs (F3); (c) PM of MSNs and Kollicoat IR (F6); (d) PM of MSNs and HPMC (F9); (e) IMC–MSNs (F3); (f) IMC–MSNs@Kollicoat IR (F6); and (g) IMC–MSNs@HPMC (F9). (**C**) IR spectra of pure IMC, IMC–SDs, and relative physical mixtures.

In order to determine the crystalline state of different kinds of SDs and physical mixture (PM) and IMC powders, PXRD analysis was performed, and the results are shown in Figure 4A. The diffractogram of pure IMC presented crystalline peaks at 10.2°, 11.6°, 16.8°, 17.0°, 19.6°, 21.8°, 26.8°, and 29.4° in the region of 5–55° (2θ) [23]. However, there were no obvious peaks in the formulation of the SDs, indicating that IMC was in the amorphous state in all of the SDs [24].

To further confirm the existing state of IMC, DSC was utilized to elucidate the thermal transitions of IMC in crystalline powder, physical mixtures, and IMC–SDs in the temperature range of 60–280 °C. As shown in Figure 4B, the corresponding curve of the pure IMC exhibited a sharp endothermic peak at around 160 °C which was associated with the melting of IMC [25]. This was also detected in the physical mixtures as a broad peak with HPMC or Kollicoat IR. However, all the SDs of IMC did not form drug crystalline peaks, indicating the IMC was completely converted to the amorphous form during the formation of solid dispersions. Thus, we can draw the conclusion that the HPMC and Kollicoat IR were added into the SDs as PIs, resulting in the amorphous form of IMC in SDs. Furthermore, the thermal analysis was based on the Gordon–Taylor equation (Equation (4)) which was used to calculate the value of *T_g_* and indicate the hydrogen bonding interaction among the SDs. According to the Gordon–Taylor equation, we can calculate the theoretical *T_g_* value of the amorphous drug system. It is reported that the measured *T_g_* value is positively deviated from the theoretical *T_g_* value, indicating that the number and intensity of hydrogen bonding for the two components in the co-polymer are stronger than those in the single polymer which is beneficial to the formation of an amorphous system [14,26]. The theoretical *T_g_* values of IMC–MSNs@Kollicoat IR (F6) and IMC–MSNs@HPMC (F9) calculated using the Gordon–Taylor equation were 141 °C and 44 °C, respectively, and the measured *T_g_* values of IMC–MSNs@Kollicoat IR (F6) and IMC–MSNs@HPMC (F9) were 197.16 ± 2.45 °C and 204.40 ± 0.33 °C, respectively. Thus, the hydrogen bonding of IMC–MSNs@HPMC (F9) was stronger than that IMC–MSNs@Kollicoat IR (F6), and all of the SDs formed an amorphous form.

(4) $$T_{g}=\frac{T_{gA}+(\left(kT_{gB}-T_{gA})W_{B}\right)}{1+\left(k-1\right)W_{B}}$$

where *W_A_* and *W_B_* are the mass fractions of copolymer A and B components, *T_g_* is the glass transition temperature, and *k* is a constant. 

Thus, we can draw the conclusion that the HPMC and Kollicoat IR were added into the SDs as PIs, and their effect of inhibiting drug crystallization was more significant according to the results of the dissolution rate and solubility analysis compared to IMC like normal formulation F3 (IMC–MSNs).

#### 3.2.3. Infrared (IR) Spectroscopy 

The IR spectroscopy analysis was performed to predict the possible interactions between IMC and carriers as well as to gain more information about the crystalline form transition of IMC inside the SDs [27]. Indomethacin (IMC) has both a carbonyl group and a hydroxyl group; therefore, it is both an acceptor of hydrogen and a donor of hydrogen. A carrier, such as MSNs, is also both an acceptor of hydrogen and a donor of hydrogen because of the silanol groups (Si–OH), but HPMC is only an acceptor of hydrogen. Therefore, IMC and different carriers of IMC–SDs are likely to have hydrogen bonding. This interaction between the carriers and IMC can be analyzed by the shift of the carbonyl peak and the hydrogen and oxygen peaks. Figure 4C shows the IR spectra of IMC and SDs, in which IMC exhibited rather broad OH vibration bands from 3400 to 2500 cm^−1^ [28]. The bands at 1692 cm^−1^, 1685 cm^−1^, and 1680 cm^−1^ originated from the stretching vibration of benzoyl ν_C=O_ of IMC, IMC–MSNs@Kollicoat IR (F6), and IMC–MSNs@HPMC (F9), respectively. In addition, this stretching region was superimposed by C–H bands (Figure 4C). Similar peaks could also be found in the other SDs or carriers, although they were a little different. The bands at 1469 cm^−1^, 1709 cm^−1^, and 1716 cm^−1^ were assigned to the deformation vibration of β C–OH and stretching vibration of the ν_C=O_ in –COOH, respectively. At the same time, the benzoyl ν_C=O_ of the physical mixture of relevant IMC–SDs were observed also at 1692 cm^−1^, suggesting that there was no interaction with the physical mixture. The results above all indicated that there were some interactions, such as hydrogen bonding, between the IMC and single carriers, including HPMC, and MSNs and binary carriers in the IMC–SDs. The formation of hydrogen bonds between the IMC and SD carriers can further maintain the supersaturation state of the solution. Weak hydrogen bonding is also one of the main factors affecting the inhibition of supersaturated crystallization.

### 3.3. In Vitro Studies

#### 3.3.1. Solubility Analysis

##### Crystalline Solubility

**Figure 5:**
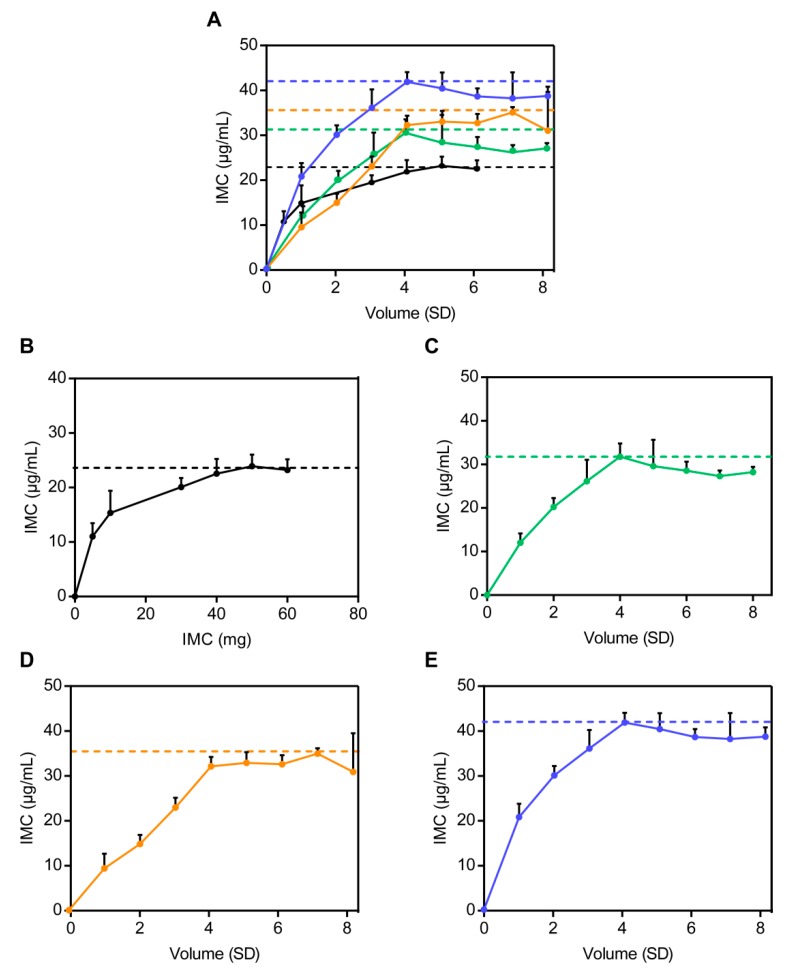
The solubility of (**A**) all of the formulations; (**B**) IMC; (**C**) IMC–MSNs (F3); (**D**) IMC–MSNs@Kollicoat IR (F6) and (**E**) IMC–MSNs@HPMC (F8). HPMC (20 µg/mL) was added to the buffer solution to inhibit the crystallization of IMC, thereby enabling the maximum release of IMC from amorphous SDs (mean ± SD, *n* = 3).

Indomethacin (IMC) has a stable crystalline solubility in pH 6.8 PBS solution with a value of 23 μg/mL (Figure 5) at 25 °C. This indicates poor solubility and low bioavailability which are characteristics of the BCS II class [29].

##### Amorphous Solubility

As determined, the solubility values of IMC–MSNs (F3), IMC–MSNs@Kollicoat IR (F6), and IMC–MSNs@HPMC (F9) were 32 μg/mL, 36 μg/mL, and 42 μg/mL, respectively, as shown in Figure 5, and the solubility of IMC for IMC–MSNs (F3), IMC–MSNs@Kollicoat IR (F6), and IMC–MSNs@HPMC (F9) were 1.4, 1.7 and 1.8 times higher than that of pure IMC as shown in Figure 5A, respectively, which means that the order of solubility was IMC–MSNs@HPMC (F9) > IMC–MSNs@Kollicoat IR (F6) > IMC–MSNs (F3) > IMC with the LLPS concentration theory (Figure 5), suggesting that the ability to enhance the solubility of crystal pure drug varies from carrier to carrier.

#### 3.3.2. In Vitro Dissolution Studies

**Figure 6:**
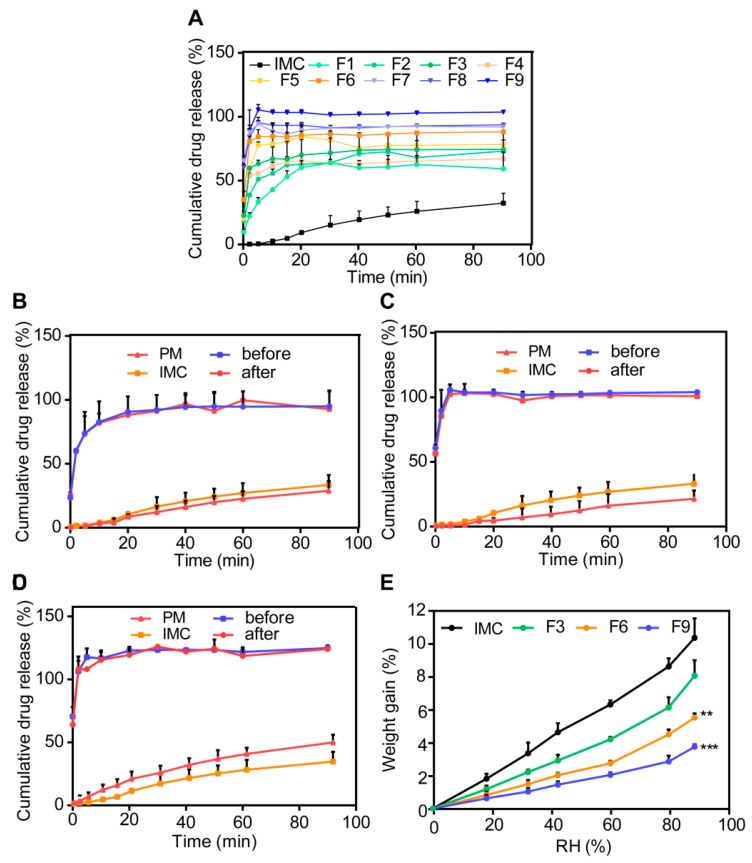
In vitro dissolution rate and moisture condition of IMC–SDs with different carriers that were stored for two months (mean ± SD, *n* = 3). (**A**) All SDs; (**B**) IMC–MSNs (F3); (**C**) IMC–MSNs@Kollicoat IR (F6); and (**D**) IMC–MSNs@HPMC (F9); (**E**) Moisture sorption properties of different IMC–SD systems: IMC–MSNs (F3); IMC–MSNs@Kollicoat IR (F6); and IMC–MSNs@HPMC (F9). Mean ± SD, *n* = 3. ** *p* < 0.01, and *** *p* < 0.001 compared to the IMC group.

The formulations selected to be subject to the dissolution rate test are listed in Table 1. We can see the release curves of all of the formulations in Figure 6A. The optimum carriers were determined based on the cumulative dissolution rate of SDs in Table 2. First, IMC–MSNs (F3), IMC–MSNs@Kollicoat IR (F6), and IMC–MSNs@HPMC (F9) were selected in the preliminary experiments because of their higher dissolution rate among all formulations with different ratios of single and binary carriers. The dissolution rate curves of IMC–MSNs (F3), IMC–MSNs@Kollicoat IR (F6), and IMC–MSNs@HPMC (F9) are shown in Figure 6B–D. The cumulative release of selected SDs was beyond 80%, while the IMC was only 20% within 1.5 h in the pH 6.8 PBS. As indicated, IMC–SDs with MSNs and PIs, such as HPMC and Kollicoat IR, played a key role in promoting the dissolution rate of IMC due to the great ability to inhibit the crystalline state of pure IMC. The IMC–MSNs@HPMC (F9) became the optimum formulation, because the stronger hydrogen bonding between HPMC and drugs form a greater ability of inhibiting precipitation [17]. In addition, based on the above results, the addition of PIs into the IMC–SDs hampered the recrystallization state of drugs so that the SDs could maintain the amorphous form for a long time via the hydrogen bonding. 

Dening et al. [9] reported that there is a dynamic adsorption equilibrium between drugs and MSNs in solid dispersions. In theory, the surface coverage of MSNs is the main factor limiting the dissolution rate of drugs [10]. Therefore, the dissolution rate reached the optimum value when the ratio of IMC to (MSNs) was 1:5 rather than other ratios. For polymers, the ability to change the dispersion state of drugs in SDs is a critical factor to decide whether it is a favorable excipient or not [30]. Therefore, if the combination of polymer as a PI and MSNs can give full play to their respective advantages, the best effect can be achieved on a single carrier.

In theory, the thermodynamic basis of SDs corresponding to the crystal form has been widely discussed [15,31,32]. The solubility enhancement in the amorphous form was calculated by the fact that the chemical potential of the amorphous form exceeded that of the crystalline form. The specific calculation equation is as follows:

(5) $$\frac{S_{A}}{S_{C}}=\exp\left(\frac{\mu_{A}-\mu_{C}}{\mathrm{RT}}\right)$$

where $\;\frac{S_{A}}{S_{C}}\;$ is the solubility ratio of the amorphous form to the crystalline form, µ_A_ and µ_C_ are the chemical potentials of the drug in the amorphous and crystalline state, respectively, R is the gas constant, and T is temperature.

However, Equation (5) is of limited use, because it only applies to systems in which drugs form an amorphous equilibrium and where drugs have minimal concentration dependence [33]. By contrast, Hansen solubility parameters (δ) may be more suitable as a preliminary indicator for showing the ability of carriers to enhance the solubility of drugs, and the equation can be expressed as follows:

(6) $$\delta^{2}=\left(\delta_{d}^{2}+\delta_{p}^{2}+\delta_{h}^{2}\right)$$

(7) $$\delta_{d}=\left(\frac{\sum_{i}\mathrm{Fdi}}{V}\right);\delta_{p}=\left(\frac{\sum_{i}F^{2}\mathrm{pi}}{V}\right);\delta_{h}=\left(\frac{\sum_{i}\mathrm{Ehi}}{V}\right)$$

where V is the molar volume, F_di_, F_pi_, and E_hi_ are contributions of functional groups to different components which refers to dispersion force, polar interaction, and hydrogen bonding, respectively [34].

It is generally believed that when the difference of δ values between two components is less than 7 MPa^1/2^, a uniform and stable phase will be produced due to the special molecular interactions [34]. The δ values of IMC, Kollicoat IR, and HPMC were 24.8 MPa^1/2^ ($\delta_{d\;}$ = 22.2 MPa^1/2^, $\delta_{p}$ = 6.0 MPa^1/2^, $\delta_{h}$ = 9.4 MPa^1/2^) [35], 32.5 MPa^1/2^ [36], and 30.6 MPa^1/2^ ($\delta_{d\;}$ = 18.0 MPa^1/2^, $\delta_{p}$ = 15.3 MPa^1/2^, $\delta_{h}$ = 19.4 MPa^1/2^) [37] based on Equations (6) and (7). Thus, the difference in solubility parameters between IMC and HPMC was calculated to be less than 7 MPa^1/2^ based on Equations (6) and (7), indicating that IMC–MSNs@HPMC (F9) had the best solubility enhancement effect. As shown in Figure 5, the same result was obtained in Figure 6, where the solubility value of IMC–MSNs@HPMC (F9) was the largest. Therefore, the obvious enhancement of the solubility in the binary carrier systems made them excellent solubilizers for BCS II class drugs, such as IMC, due to the massive hydroxyl groups of the HPMC and Kollicoat IR in SDs [38]. 

#### 3.3.3. Moisture Sorption Analysis

Theoretically, the water sorption isotherm for physical mixtures is equal to the sum of isotherms of single component isotherms. However, the amount of water absorption in SDs is not a simply linear addition to that of in individual components thanks to molecular interactions between the drug and carriers [39]. Therefore, the water absorption condition can provide interactions between the drug and carriers in SDs, which can be considered as an assistant evaluation factor of stability.

Moisture sorption of different SDs is shown in Figure 6E. It is indicated that SDs with different carriers have different moisture absorption ability. The weight gain values of IMC–MSNs (F3), IMC–MSNs@Kollicoat IR (F6), and IMC–MSNs@HPMC (F9) showed 0.82, 0.44, and 0.32 folds compared to IMC, respectively, which was consistent with the in vitro dissolution experiment. It is reported that the moisture sorption ability of SDs is relative to prediction based on the weighted contribution of drugs and polymers, and that the hygroscopic capacity of solid dispersions is reduced due to the interaction between the polymer molecules and the drug [40].

#### 3.3.4. Stability Studies

The results showed that the dissolution rate of SDs (i.e., IMC–MSNs, IMC–MSNs@Kollicoat IR, and IMC–MSNs@HPMC) stored for two months at 25 °C and 60% RH was similar to that of freshly prepared SDs. In addition, the favorable results of the water vapor sorption experiment were similar with the results of the in vitro release experiment, indicating acceptable physical stability.

Ueda et al. [40] has reported that solid dispersion with a higher drug content was less stable at 40 °C and 75% RH over three months. According to the research, it is suggested that the specific intermolecular interaction of the binary carriers contributes to the high physical stability. Otherwise, the stability of SDs can be affected by crystallization. The crystalline growth of IMC is controlled by the diffusion of molecules and the hydrogen bonding of IMC and carriers [41]. Above all, the IMC–MSNs (F3), IMC–MSNs@Kollicoat IR (F6), and IMC–MSNs@HPMC (F9) can form optimum interactions to overcome the instability factors of SDs.

#### 3.3.5. Permeability and Bioavailability Studies

The parallel artificial membrane permeability assay (PAMPA) is composed of an artificial membrane, such as dodecane, which is composed of two compartments including a donor and an acceptor place [20]. Different measurements can obtain various values of PAMPA permeability coefficients, just like the so-called percentage of flux (%F) or transported solute (%T) which measures the part of the test compound in the acceptor compartment. The PAMPA is able to rapidly determine the tendency of compounds to penetrate membranes by passive diffusion and is therefore suitable for screening solid dispersion and relative drugs [22]. 

**Figure 7:**
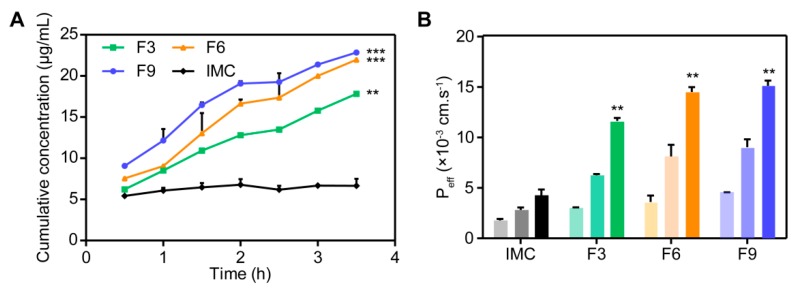
(**A**) Permeation profile of pure IMC and IMC–SDs in parallel artificial membrane permeability assay (PAMPA) experiment (mean ± SD, *n* = 3). (**B**) Permeation effective of IMC and IMC–SDs at 1.5 h, 2.5 h, and 3.5 h with the color becoming darker, respectively (mean ± SD, *n* = 3). ** *p* < 0.01, and *** *p* < 0.001 compared to the IMC group.

The results are shown in Figure 7A,B. The initializing concentration of IMC was 23 μg/mL, and we can see that the amount of permeated IMC in SDs was greatly increased compared to pure IMC as a control group. The cumulative permeation of IMC in SDs like IMC–MSNs@HPMC (F9), IMC–MSNs@Kollicoat IR (F6), and IMC–MSNs (F3) reached 22.8, 21.9, and 17.8 μg/mL, respectively, in 3.5 h. Moreover, the *P_eff_* values also confirmed the significantly improved permeation effect of IMC SDs. It is suggested that solid dispersion could improve the permeability of IMC due to the fact of its smaller size and more uniform dispersion state. In addition, the IMC–MSNs@HPMC (F9) reached an optimum permeated value because of the stronger hydrogen bonding interaction and higher in vitro dissolution rate of MSNs and HPMC binary polymer system. According to the reported NMR and molecular dynamics studies [42], IMC–SDs were able to penetrate into lipid membranes, suggesting a sharp increase in oral bioavailability of IMC–SDs.

### 3.4. In Vivo Studies

#### 3.4.1. In Vivo Pharmacokinetic Study

**Figure 8:**
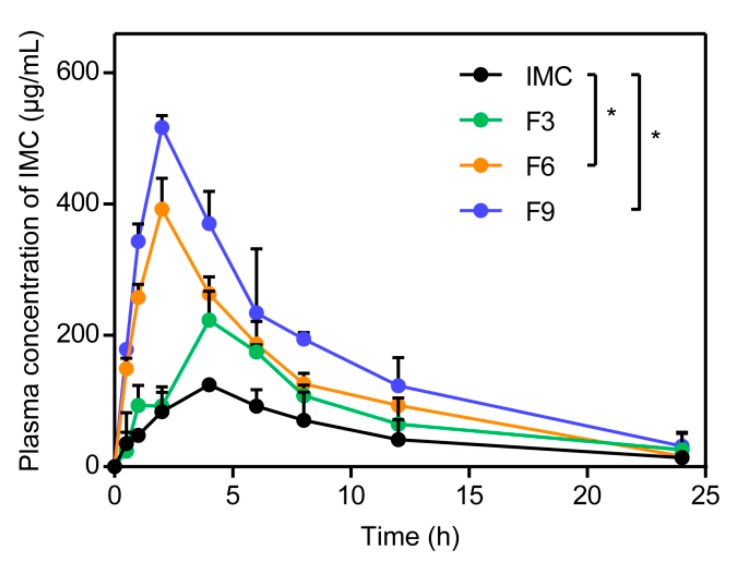
Mean drug concentration-time profiles of IMC, IMC–MSNs (F3), IMC–MSNs@Kollicoat IR (F6), and IMC–MSNs@HPMC (F9) in rats after oral administration (mean ± SD, *n* = 3). Unpaired Student’s *t*-test was used to do statistical analysis. Statistical significance was set as * *p* < 0.05, compared to the IMC group.

The in vitro pharmacokinetic results are shown in Figure 8 followed by the oral delivery of IMC–SDs. The various pharmacokinetic parameters are shown in Table 3. After oral administration of IMC–SDs, it was obvious that the bioavailability of IMC–MSNs (F3), SDs with IMC–MSNs@Kollicoat IR (F6), and IMC–MSNs@HPMC (F9) were all improved compared to pure IMC. The value of the area under the plasma concentration–time curve (AUC) of IMC–MSNs (F3), IMC–MSNs@Kollicoat IR (F6), and IMC–MSNs@HPMC (F9) were approximately 1.6 fold, 2.4 fold, and 3.3 fold higher than pure IMC, respectively. At the same time, the C_max_ for IMC–MSNs (F3), IMC–MSNs@Kollicoat IR (F6), and IMC–MSNs@HPMC (F9) were approximately 2 fold, 3 fold, and 4 fold higher than pure IMC, respectively. In addition, both the values of AUC and C_max_ for the formulation of SDs with MSNs and HPMC (F9) were optimum. It is reported that HPMC as a PI has a greater ability to inhibit the drug from forming a recrystallization state so that the drug can maintain the amorphous form in the blood circulation in the body and more of the drug can be released more easily to reduce the suffering of the patient compared to other SDs. Therefore, the dissolution-enhanced ability of IMC–MSNs (F3), IMC–MSNs@Kollicoat IR (F6), and IMC–MSNs@HPMC (F9) all can improve the bioavailability of IMC, and IMC–MSNs@HPMC (F9) was the best formulation among all of the SDs.

#### 3.4.2. In Vivo Gas Intestine Tract Absorption Studies

**Figure 9:**
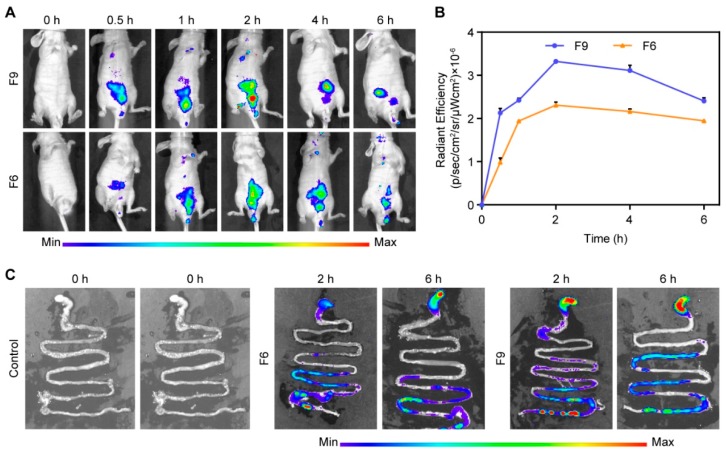
In vivo absorption of IMC–SDs. (**A**) Representative in vivo images of mice after gavage with IMC–SDs labeled IR820. Color scale: Minimum = 2.12 × 10^6^; Maximum = 1.00 × 10^8^. (**B**) Quantitative fluorescent intensity of IMC–SDs (mean ± SD, *n* = 3). Average radiant efficiency units: (p/s)/(µW/cm^2^). (**C**) Representative in vivo images of the stomach and small intestine of mice after gavage with IMC–SDs labeled IR820. Color scale: Minimum = 2.12 × 10^6^; Maximum = 1.00 × 10^8^.

To further study the influence of different PIs added to the IMC–SDs for the oral absorption of drugs, the in vivo imaging system was applied to observe the gas intestinal tract absorption of IMC- MSNs@Kollicoat IR (F6) and IMC–MSNs@HPMC (F9). The in vivo absorption results are shown in Figure 9 followed by the oral delivery of IMC–SDs. Firstly, the biodistribution of SDs was observed after a single oral dosing of the control group and test group in Balb/c mice. The mice were sacrificed at 2 and 4 h after the oral dosing, and their excised intestines were imaged for fluorescent signal using an in vivo imaging system (IVIS). The fluorescent signal intensity areas of the stomach, small intestine, and colon are shown in Figure 9C. After 30 min, the test group SDs began to move through the stomach into the front of the small intestine gradually. Over the next hours, the signal increased in the part of small intestines and different absorption trend appeared. It was obvious that IMC–MSNs@HPMC (F9) presented a significantly larger fluorescent area than that of other SDs, suggesting that a large amount of IMC–MSNs@HPMC (F9) entered into the gastrointestinal (GI) tract. The same result was shown at Figure 9B. Finally, the signal was eliminated from the GI tract after 6 h which was the same as that at 4 h.

From the IVIS imaging and fluorescent quantity values, different absorption conditions of SDs in the GI tract are clearly presented in the Figure 9. It is well known that the small intestine plays a critical role in the process of drug action, because it has a large surface that uptakes drugs and nutrients and prevents the gut lumen micro biota from entering at the same time [43]. However, pure IMC cannot be absorbed effectively due to the fact of its poor solubility and even causes severe adverse effects. Herein, various fabricated IMC–SDs with different molecular interactions between the carriers and IMC in the SDs can achieve different effects which are consistent with in vitro dissolution results, but they all can be used to improve the compliance of patients [44]. 

## 4. Conclusions

This study manifested a feasible scheme using the solvent evaporation method to understand the amorphous form of IMC–SDs with MSNs. The different precipitation inhibitors, including HPMC and Kollicoat IR, were added into the IMC–SDs to maintain the amorphous form. The optimal formulation was selected by a dissolution experiment which greatly improved the amorphous solubility of IMC. The obtained results suggested that IMC–SDs prepared by MSNs as a carrier with the addition of Kollicoat IR or HPMC as precipitation inhibitors improved the in vitro dissolution rates by approximately 56% and 77% respectively compared to the IMC-MSNs. The IMC–SDs in the presence of MSNs and Kollicoat IR or HPMC as PIs improved the in vivo bioavailability of IMC by approximately 241% and 331%, respectively. Moreover, the images of IR, DSC, and PXRD confirmed the amorphous form of SDs as indicated in the moisture absorption experiment. Also, IMC–SDs showed a similar dissolution rate before and after being stored at 25 °C for two months, demonstrating their favorable stability. In summary, the SDs formulated by the HPMC and MSN binary system have promising prospects for improving solubility and bioavailability and provide great insights into effective oral drug delivery. The addition of precipitation inhibitors will play a crucial role in improving the bioavailability of drugs in SD systems.

## Figures and Tables

**Table 1:** Different ratios (% *w/w* ratio) of drug and carriers (i.e., polymers and MSNs) of IMC solid dispersions.

Formulation	IMC	MSNs	Kollicoat IR	HPMC
F1	1	1	-	-
F2	1	3	-	-
F3	1	5	-	-
F4	1	1	3	-
F5	1	3	3	-
F6	1	5	3	-
F7	1	1	-	3
F8	1	3	-	3
F9	1	5	-	3

IMC, indomethacin; MSNs, mesoporous silica nanoparticles; HPMC, hydroxypropyl methylcellulose.

**Table 2:** Cumulative dissolution rate of different IMC–SDs at 90 min prepared with different carriers IMC–MSNs (F3), IMC–MSNs@Kollicoat IR (F6), IMC–MSNs@HPMC (F9) and increased folds compared to pure IMC (mean ± SD, *n* = 3).

Formulation	Cumulative Drug Dissolution Rate (%)	Folds
IMC	27.43 ± 0.96	1.00 ± 0.28
F3	74.96 ± 12.43	2.73 ± 0.45
F6	83.54 ± 0.39	3.23 ± 0.02
F9	104.00 ± 1.39	3.79 ± 0.05

**Table 3:** Pharmacokinetic parameters of IMC after oral administration of different IMC–SDs at a dose of 40 mg/kg to rats (mean ± SD, *n* = 3).

Formulation	*C_max_* (μg/mL)	*T_max_* (h)	AUC_0__→t_ (μg·mL^−1^·h)	Frel (%)
IMC	124.6 ± 3.8	4.0	1236.5 ± 188.8	-
F3	223.1 ± 44.1	4.0	2010.5 ± 441.9	162.6 ± 35.7
F6	392.5 ± 47.0	2.0	2976.0 ± 207.9	240.7 ± 16.8
F9	516.8 ± 18.1	2.0	4086.5 ± 485.8	330.5 ± 39.3
